# Priority determines *Tribolium* competitive outcome in a food-limited environment

**DOI:** 10.1371/journal.pone.0235289

**Published:** 2020-07-09

**Authors:** Zane Holditch, Aaron D. Smith

**Affiliations:** 1 Department of Biological Sciences, Northern Arizona University, Flagstaff, AZ, United States of America; 2 Department of Entomology, Purdue University, West Lafayette, IN, United States of America; US Department of Agriculture, UNITED STATES

## Abstract

Flour beetles are a classic model system for studying competitive dynamics between species occupying the same ecological niche. Competitive performance is often interpreted in terms of biological species traits such as fecundity, resource use, and predation. However, many studies only measure competitive ability when species enter an environment simultaneously, and thus do not consider how the relative timing of species’ arrival may determine competitive outcome (*i*.*e*., priority effects). Whether priority effects may influence competition in *Tribolium* remains to be tested. The present study examined the importance of priority effects in competitions between two common species of flour beetle (Coleoptera: Tenebrionidae): *Tribolium castaneum* and *T*. *confusum*. To investigate whether priority effects confer competitive advantages to *Tribolium* beetles, relative introduction times of *T*. *castaneum* and *T*. *confusum* to competitive arenas were manipulated, and adult populations were measured for seven months. Four important patterns were noted: (1) *Tribolium* species given two-weeks priority access to experimental arenas attained larger populations than their late-arriving competitor, (2) when founding adults were introduced simultaneously, *T*. *castaneum* was competitively dominant, (3) *T*. *castaneum* benefited more from priority arrival than *T*. *confusum*, and (4) available bran resources largely predicted population decline in adult beetles toward the end of the experiment. These results suggest competitive outcome in *Tribolium* is not always predicted by species’ identity, and that performance could instead be determined by the timing of species’ arrivals and available resources.

## Introduction

*Tribolium* flour beetles (Coleoptera: Tenebrionidae) are excellent model organisms for testing hypotheses in genetics and population ecology [1–3], especially ecological competition. Thomas Park, in his classic series of experiments [4–6], was first to demonstrate that direct competition between *T*. *castaneum* (Herbst) and *T*. *confusum* (duVal) could cause the elimination of one of the two species. Despite some level of unpredictability in outcome [7], Park [4,5] revealed that competitive outcome in *Tribolium* could be influenced by adjusting initial environmental conditions, such as humidity and temperature. Park’s experiments suggested competitive performance is not intrinsic to a species, and that the outcome of interspecific competition depends upon the initial conditions of an ecological space.

More recent investigations of animal competition emphasize the importance of historical contingency in predicting competitive performance and community assembly [8,9]. Species’ migration to a community may be affected by differences in reproductive phenology or seasonal renewal of resources [10]. Thus, founding individuals may enter an environment in sequence, thereby modifying the intensity of interspecific competition and shaping community composition as a result [11,12]. Early-arriving species often experience reduced competitive pressures compared to late-arriving species [13], and so some species appear to have evolved asynchronous breeding times to delay the onset of direct competition [10]. When individuals benefit from early-arrival (*i*.*e*., “priority effects”), competitive performance appears to be determined by both the relative order of arrival and the amount of time between the arrivals of the interacting species [14–17]. Priority effects appear to mediate interspecific competition in many systems including plant communities [18], ectomycorrhizal fungi [19], and insects [20–22]. However, despite the importance of *Tribolium* beetles in competition research, priority effects in this system have not been measured.

Experiments involving *Tribolium* have strongly supported the role of competitive interactions in structuring ecological communities [23]. This research has revealed competitive performance in *Tribolium* species may be influenced by behavioral and ecological characteristics such as reproductive interference [24], intraguild cannibalism [25] as well as disease, food quality, and fecundity within the experimental climate [4,5,26]. However, much of this work examines competition when species are introduced to an environment at the same time, which makes it difficult to determine the importance of these drivers when arrival order in natural environments is sequential. For instance, if differences in arrival order also impact competitive interactions, then the importance of environment and species traits in determining competitive outcome could be mitigated. That is, early-arrival could allow a competitively weaker species to resist invasion, even when environmental conditions might already favor the invading species. In the present experiment, we attempt to investigate whether priority establishment could drive competitive outcomes between *T*. *confusum* and *T*. *castaneum* under identical environmental conditions. The following predictions were made: (1) if priority effects determine interspecific competition more so than species identity, reversing the order of species arrival should reverse the outcome; and (2) when competitors arrive simultaneously, competitive outcome will be determined by how species characteristics (*e*.*g*., resource use, fecundity, predation, etc.) are expressed within the experimental environment. This study contributes to a growing body of research that emphasizes the role of historical contingency in animal competition and community assembly.

## Materials and methods

### Study species

*Tribolium castaneum* (Herbst) and *T*. *confusum* (duVal) are widely used in population ecology research due to their ease of culture in laboratory conditions and their economic importance as common grain pests [27]. Consequently, much is known about the genetic relationships and life history traits of the two species. *T*. *castaneum* and *T*. *confusum* are more genetically related to each other than to any other member of the *Tribolium* genus [28,29]. The two species are also highly ecologically similar, as they both form natural populations in milled cereals [30]. Both species utilize stored grain products as a food source and as a living space, and appear to have done so for more than 4,000 years [31].

Fecundity and generation time in *Tribolium* appears to be highly sensitive to relative humidity (R.H.) and temperature, though optimal living conditions differ slightly between species [4,5]. Both species require approximately one month to complete a single generation (*i*.*e*., egg to adult), and may lay 2–16 eggs per day [32]. In favorable conditions, where food is not limiting, adult beetles may live for more than three years [33], though the average lifespan is roughly six months [34]. Development in *T*. *castaneum* is fastest in warm and humid environments (35–37.5 ºC and >70% R.H.), whereas *T*. *confusum* performs best in slightly cooler conditions (32.5 ºC and 70% R.H) [35,36]. It is important to carefully control ambient climate when maintaining populations, as certain conditions may favor a single species in competition experiments. In the present experiment, beetle populations were maintained at 26 ± 1°C and 55 ± 5% R.H., which is similar to temperature and humidity conditions in previous *Tribolium* competition studies [27].

### Experimental design

Colonies were established for *Tribolium castaneum* and *Tribolium confusum* using 200 stock adults purchased from Ward’s Science, Rochester NY. Seven months before trials, beetles were kept in a special bran medium which had been conditioned to 26 ± 1°C and 55 ± 5% R.H. All beetles were kept in medium consisting of eight parts bran, four parts flour, four parts brewer’s yeast, and one part Fluker’s Cricket supplement (although 95% flour medium is standard in most *Tribolium* studies, 27).

To test the effect of priority establishment on competition between *T*. *castaneum* and *T*. *confusum*, beetles were assigned to three treatments in which relative introduction times were manipulated for each species, and two single-species populations as controls. Each treatment consisted of ten competitive arenas (5 x 5.5 x 3 cm Tupperware enclosures) containing an initial five g of medium (see above for proportions). In single-species populations, 20 adult beetles (*T*. *castaneum* in CS Solo; *T*. *confusum* in CF Solo) were introduced to ten arenas and allowed to colonize the experimental medium without any interspecies interactions. In priority treatments, species arrival was manipulated by allowing a prior population of ten adults of either species to remain isolated until introducing ten adults of the second species two weeks later. In CF Priority, *T*. *confusum* was introduced the experimental medium first, whereas in CS Priority, *T*. *castaneum* was given early arrival. Immediate competition of *Tribolium* was also examined, when ten adults of both species were added to the arenas at the same time (i.e., Treatment: Simultaneous Arrival). Thus, once trials began, all 50 arenas contained an initial combined total of 800 adult beetles, and 200 beetles added two weeks later. All beetles were selected at random from the stock colonies, without preference for age or sex, and maintained at the environmental conditions described above throughout the experiment. Every 30 days, (i.e., month), live adult beetles were sifted from the medium, weighed, counted, and identified to species. Adult cadavers were also counted and identified when possible. Live and dead adults and larvae were then returned to the arenas along with the medium. Populations were measured seven times during the experimental period (*i*.*e*., Month 1–7). Population measurements ceased when adult *T*. *castaneum* populations collapsed in all treatments during the final months of the study.

Finally, monthly resource use between *Tribolium* species was also estimated by measuring the mass of the residual medium, which also contained unidentified eggs and larvae. Substrate was returned to the arena after each census. Fresh bran substrate was not added at any time during the experiment.

### Statistical analysis

A generalized linear model (negative-binomial error structure with log link function) was used to measure the effects of treatment, beetle species, and their interactions on the adult beetle populations. If fixed-effects were significant overall, *post-hoc* tests were performed to assess significant differences between treatment means. *Tribolium* species were considered to be competitively successful if their average adult population was significantly larger than its competitor species during the experimental period.

Adult populations appeared to collapse in treatment groups between month six and seven the experiment. To examine whether declining substrate levels impacted survivorship in beetle populations, a three-factor ANOVA was used to examine the effect of treatment, beetle species, net substrate loss, and their three-way interactions on the proportion of adults in month seven relative to month six (i.e., Survivorship). Prior to the analysis, values of survivorship were transformed using a log(x+1) transformation. To determine relevant factors in the model, non-significant effects were eliminated in a stepwise manner using a Bonferroni-adjusted threshold for significance (α = 0.0071). If treatment significantly affected survivorship, a *post hoc* Tukey HSD test was used to examine differences in average beetle survivorship between each treatment. If net substrate loss significantly affected survivorship, a simple linear-regression was used to assess the relationship between substrate levels and beetle survivorship.

Finally, a repeated measures linear mixed-model was used to estimate bran consumption by beetles during single-species trials. *Tribolium* species and month (1–7) were treated as fixed-effects of substrate mass (g). Each population (i.e., “POP_ID”) was fitted as a random effect. All statistical tests were performed in JMP Pro [37]. Means and ± standard errors are reported for adult beetle populations throughout.

## Results

**Fig 1:**
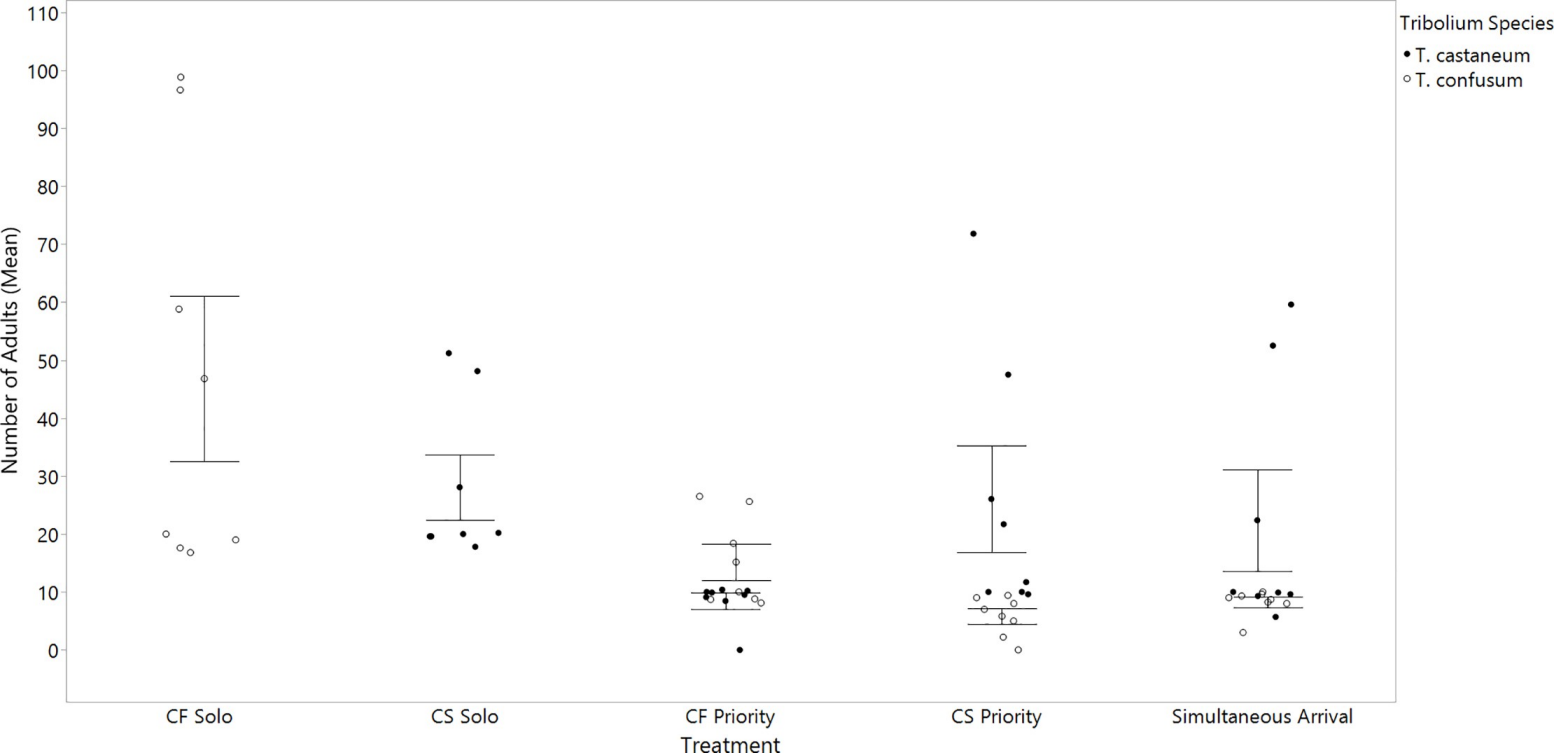
Mean adult populations of *T*. *castaneum* and *T*. *confusum* in each of the treatment groups. Treatment significantly affected adult populations of *T*. *castaneum* and *T*. *confusum* (p<0.0001). Error bars are constructed using one standard error from the mean.

On average, beetle colonies contained 21.8 ± 2 adults during the seven-month experimental period, with an average of 1744 ± 395 individuals counted per month. Mean population size between the two species in all treatment groups was approximately the same (21.2 ± 1.2 in *T*. *castaneum*; 19.0 ± 1.5 in *T*. *confusum*). An overall significant effect of treatment (χ^2^_1_ = 296.33, p<0.0001) and beetle species (χ^2^_1_ = 35.22, p<0.0001) on adult *Tribolium* populations was supported by the generalized linear model. An interaction between beetle species and treatment was also supported by the analysis (χ^2^_3_ = 240.96, p<0.0001). Thus, both *T*. *castaneum* and *T*. *confusum* populations were not equally affected by treatments (Fig 1). Parameter estimates from the mixed model are described in Table 1. Important species differences and demographic trends are described below.

**Table 1:** Generalized linear model parameter estimates.

Parameter	Estimate	Std. Error	95% Wald Confidence Interval		Hypothesis Test		
			Lower	Upper	Wald Chi-Square	df	Sig.
	3.85	0.09	3.67	4.03	1748.07	1	**0**
**(Intercept)**	-1.13	0.11	-1.35	-0.9	97.52	1	**0**
**[Treatment = CF Priority]**	-2.09	0.12	-2.32	-1.86	312.2	1	**0**
**[Treatment = CS Priority]**	-1.74	0.1	-1.94	-1.54	300.01	1	**0**
**[Treatment = Solo]**	-0.51	0.11	-0.73	-0.29	20.61	1	**0**
**[Beetle Species = T. confusum]**	-0.07	0.14	-0.35	0.2	0.27	1	0.6
[Treatment = CF Priority] * [Beetle Species = T. confusum]	2.01	0.18	1.66	2.37	123.77	1	**0**
[Treatment = CS Priority] * [Beetle Species = T. confusum]	1.51	0.17	1.18	1.84	79.72	1	**0**

Dependent Variable: Adult Population

Model: (Intercept), Treatment, Beetle Species, Treatment * Beetle Species

### Single-species populations

**Fig 2:**
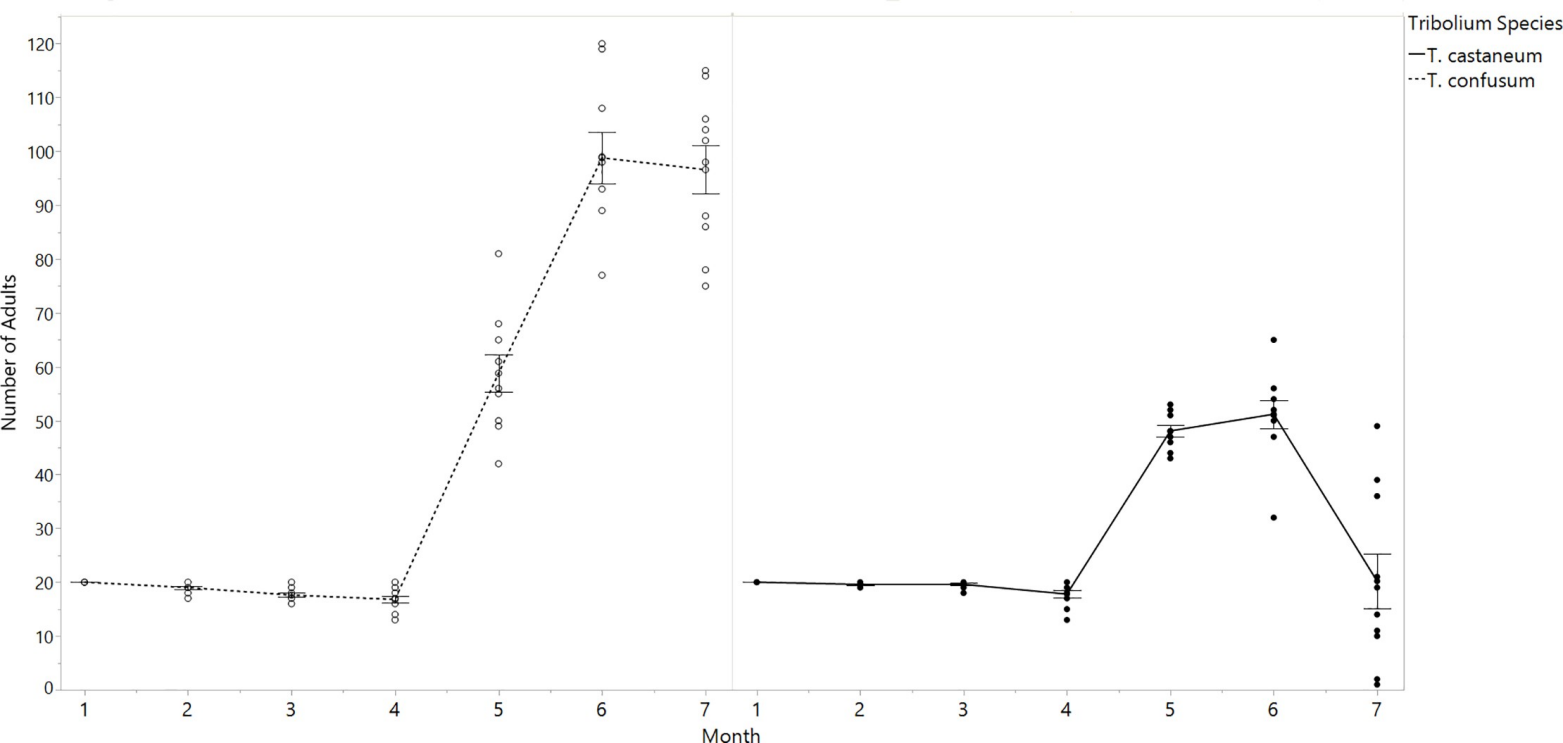
Monthly population of *T*. *castaneum* and *T*. *confusum* in single-species treatments. Peak adult *T*. *confusum* population (month six) was 88% higher than *T*. *castaneum* (p<0.0001). Prior to the end of the experiment (month six), *T*. *castaneum* populations markedly declined, while *T*. *confusum* populations remained unchanged relative to the previous month.

In single-species populations, both *T*. *confusum* and *T*. *castaneum* attained populations >20 adults after four months (Fig 2). Mean *T*. *confusum* populations were much higher than *T*. *castaneum* during single-species trials (p<0.0001). Over the seven month experimental period, *T*. *confusum* single-species populations contained an average of 46.8 ± 2.5 adults, whereas *T*. *castaneum* had only 28.1 ± 2.5 adults (Fig 2). However, by month seven, *T*. *castaneum* adults populations declined by 60.8. ± 9.4% relative to their population size in the previous month.

### Simultaneous arrival

**Fig 3:**
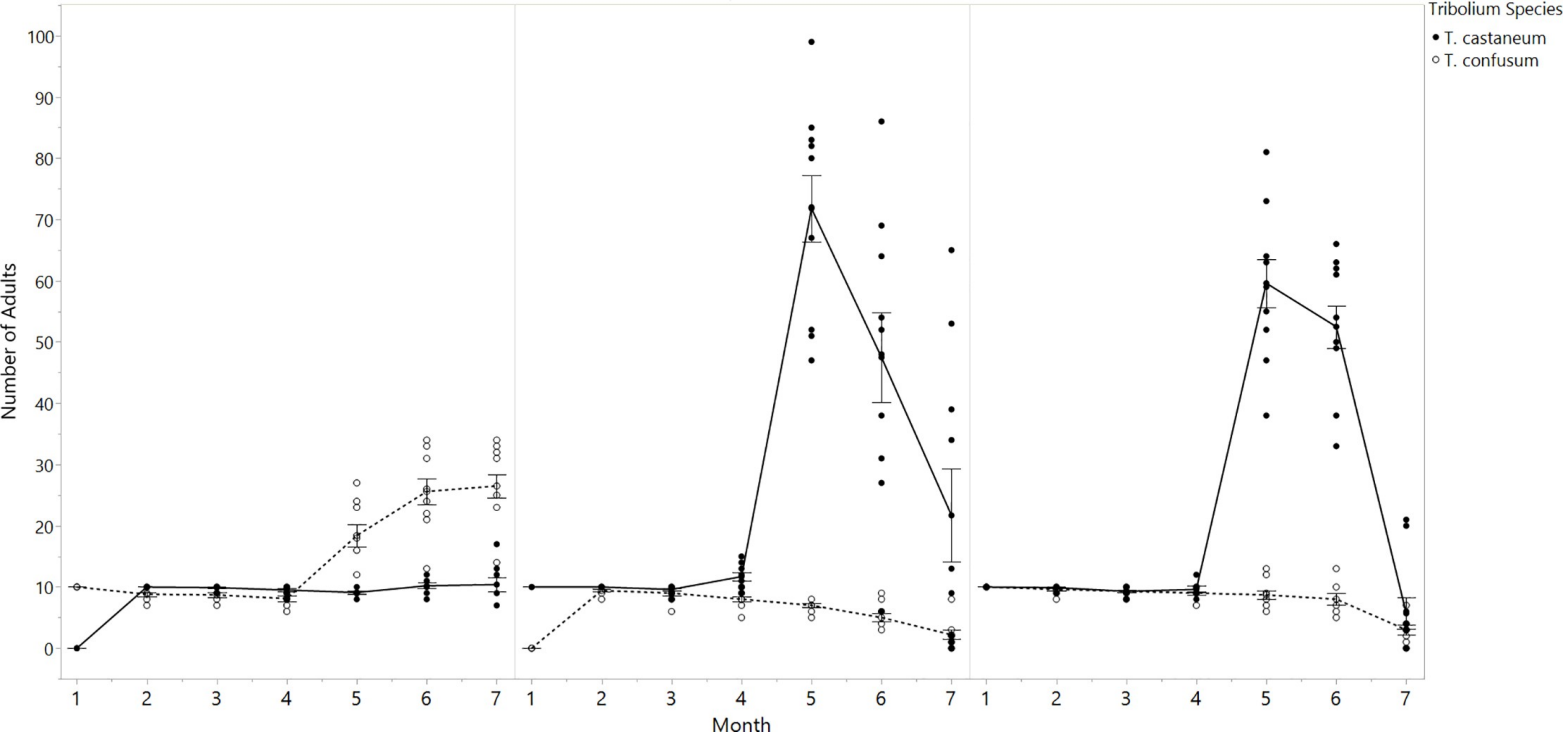
Adult populations of *T*. *castaneum* and *T*. *confusum* under interspecies competition. (A) Monthly populations of *T*. *castaneum* in response to the two-week early arrival of *T*. *confusum* (B) Monthly populations of *T*. *confusum* after the introduction of *T*. *castaneum* two-weeks prior. (C) Adult populations of *T*. *castaneum* and *T*. *confusum* following simultaneous introduction to arenas. Error bars are constructed using one standard error from the mean.

When founding adults were introduced simultaneously, *T*. *castaneum* attained significantly larger mean populations than *T*. *confusum* (p<0.0001, Fig 3). As in all previous treatments, both species populations coexisted with 10 ± 1 individuals until *T*. *castaneum* reached higher population sizes than *T*. *confusum* after month four, when adult populations increased by a factor of 6.2. However, following month five, *T*. *castaneum* populations collapsed by 88 ± 6%, similar to single-species and priority trials.

### Priority competition

*Tribolium* species that had received priority arrival attained larger adult populations compared to their competitor species. When *T*. *confusum* was introduced two-weeks early, average adult populations were approximately 2.6 times larger than coexisting *T*. *castaneum* populations (p<0.0012, Fig 3). *T*. *confusum* supported an average population size of 15.2 ± 1 adults, whereas *T*. *castaneum* populations contained an average of 8.3 ± 0.4 adults. Hence *T*. *castaneum* populations did not appear to recruit additional members beyond the 10 founding adults. In *T*. *confusum*, adult populations were >10 by month five, which is consistent with patterns in single-species treatments. Populations of *T*. *castaneum* were also smaller compared to single-species populations, and grew by only 11% in month five. Unexpectedly, however, CF Priority is the only treatment in which *T*. *castaneum* populations did not appear to collapse between month six and seven (Fig 3).

When *T*. *castaneum* arrived two-weeks early, all 10 trials supported significantly higher average *T*. *castaneum* populations than *T*. *confusum* (p<0.0001, Fig 3). Average population size of *T*. *castaneum* was 26 ± 3.2 adults, which was 4.4 times larger than *T*. *confusum* (5.8 ± 0.4 adults).

### Population growth and substrate consumption

**Fig 4:**
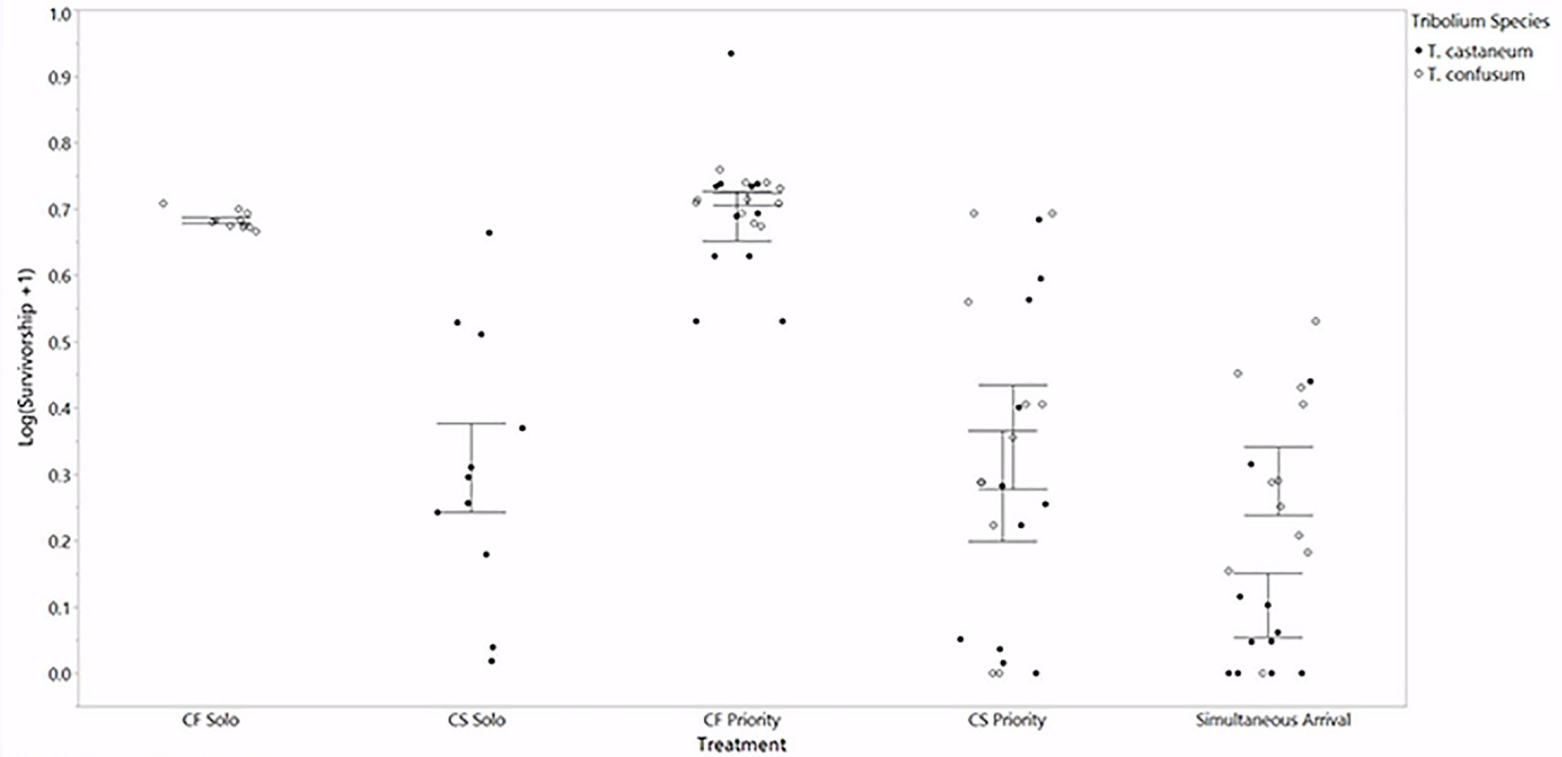
Survivorship in adult beetle populations during the last two months of the experiment. Survivorship was more strongly predicted by treatment (p<0.0001) then beetle species. Error bars are constructed using one standard error from the mean.

According to a three-factor ANOVA, the proportion of surviving adult beetles counted during the last two months of the experiment varied between treatments (F_12, 67_ = 16.2, p<0.0001, Table 2). Additionally, two-way interactions between net substrate loss and treatment (F_4, 67_ = 5.36, p = 0.0008), and three-way interactions between treatment, beetle species, and net substrate loss (F_4, 67_ = 4.08 p = 0.0051, Table 2) were supported by the model. Therefore, while average survivorship across all treatments did not differ significantly between species, *T*. *confusum* and *T*. *castaneum* survivorship was differentially impacted by net substrate loss and treatment. A *post hoc* Tukey analysis (Fig 4) revealed average beetle survivorship was lowest in *T*. *castaneum* solitary populations (0.31 ± 0.056) and when beetle were added to arenas at the same time (0.20 ± 0.069). Survivorship was highest in CF Priority and CF Solo (0.70 ± 0.089 and 0.68 ± 0.053, respectively). Survivorship in CF Solo was significantly higher than CS Solo (p<0.0001), CS Priority (p = 0.0004), and when beetles were added simultaneously to arenas (p = 0.0158). Adult survivorship was also significantly higher in CF Priority, relative to CS Solo (p<0.0016) and CS Priority (p<0.0342). In all other treatment comparisons, beetle survivorship was approximately even between species (see Fig 4).

**Table 2:** Parameter estimates for survivorship ANOVA.

Term	Estimate	Std Error	t Ratio	Prob>|t|
**Intercept**	0.48	0.03	16.98	**< .0001**
**Treatment[CF Priority]**	0.2	0.07	2.62	**0.0110**
**Treatment[CF Solo]**	0.2	0.05	4.02	**0.0001**
**Treatment[CS Priority]**	-0.09	0.04	-2.21	**0.0309**
**Treatment[CS Solo]**	-0.22	0.05	-4.31	**< .0001**
Treatment[CF Priority]*(Net Substrate Loss (Initial—Final)-1.89523)	-0.07	0.26	-0.29	0.7763
Treatment[CF Solo]*(Net Substrate Loss (Initial—Final)-1.89523)	1.21	0.41	2.97	**0.0041**
Treatment[CS Priority]*(Net Substrate Loss (Initial—Final)-1.89523)	-0.63	0.21	-2.98	**0.0040**
Treatment[CS Solo]*(Net Substrate Loss (Initial—Final)-1.89523)	1.01	0.41	2.48	**0.0156**
Treatment[CF Priority]*Beetle[T. castaneum]*(Net Substrate Loss (Initial—Final)-1.89523)	0.03	0.1	0.29	0.7747
Treatment[CF Solo]*Beetle[T. castaneum]*(Net Substrate Loss (Initial—Final)-1.89523)	1.23	0.41	3.02	**0.0036**
Treatment[CS Priority]*Beetle[T. castaneum]*(Net Substrate Loss (Initial—Final)-1.89523)	-0.44	0.17	-2.54	**0.0135**
Treatment[CS Solo]*Beetle[T. castaneum]*(Net Substrate Loss (Initial—Final)-1.89523)	-0.3	0.41	-0.74	0.4591

**Fig 5:**
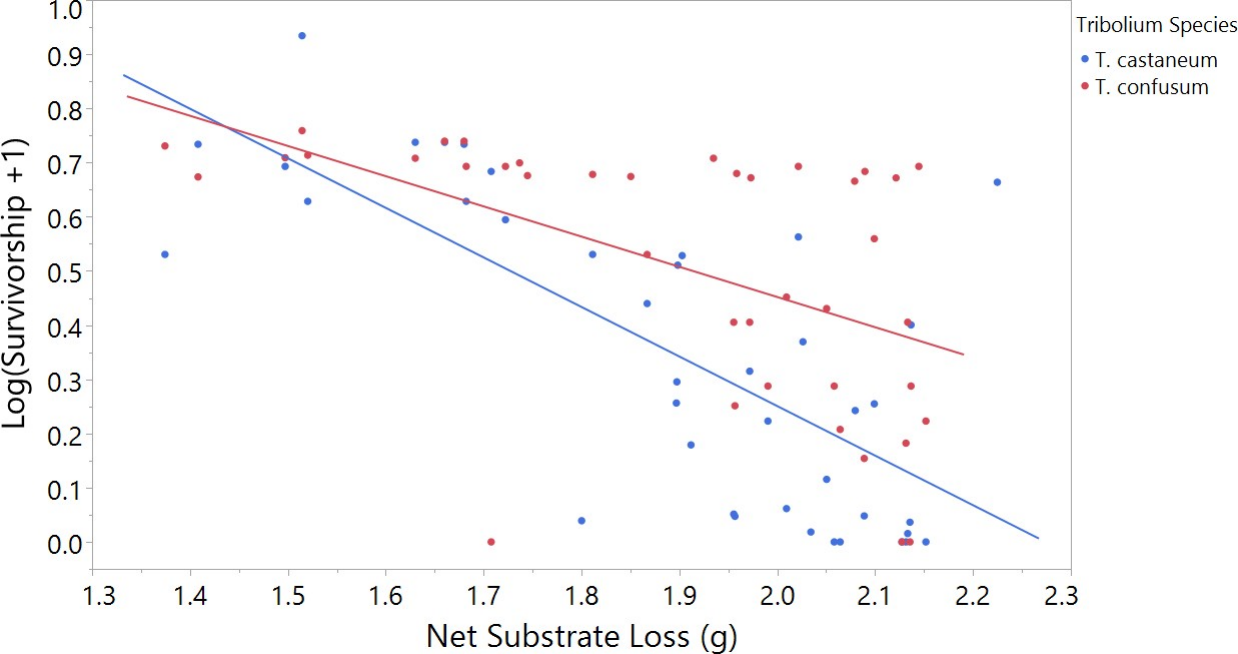
Adult survivorship during the remaining two months of trials as a function of the total amount of bran substrate consumed during the seven month period. Net substrate loss (g) on the horizontal axis begins at 1.3 g to display the variation in beetle survivorship. Beetle species appeared to be differentially affected by substrate loss (F_4, 67_ = 4.08, p = 0.0051). Total substrate loss (g) accounted for 51% of the variation in *T*. *castaneum* survivorship (F_1, 38_ = 41.7, p<0.0001), and 27% of the survivorship variation in *T*. *confusum* (F_1, 38_ = 15.1, p = 0.0004).

A simple linear regression indicated beetle survivorship was negatively impacted by net substrate loss (F_1, 7 8_ = 44.9, p<0.0001, R^2^_adj_ = 0.36). When comparing beetle survivorship between species, net substrate loss explained 51% of the variation in *T*. *castaneum* survivorship (F_1, 38_ = 41.7, p<0.0001, R^2^_adj_ = 0.51, Fig 5), and 27% of the survivorship variation for *T*. *confusum* (F_1, 38_ = 15.1, p = 0.0004, R^2^_adj_ = 0.27, Fig 5).

Lastly, during single-species trials, bran substrate gradually declined by month (F_1, 118_ = 4807.9, p<0.0001), according to a mixed model analysis. However, substrate disappeared more rapidly in *T*. *castaneum* populations than *T*. *confusum* (F_1, 18_ = 16.6, p = 0.0007), despite *T*. *castaneum* attaining smaller average populations (see Fig 1).

## Discussion

In the present study, timing of arrival was manipulated in competitions between *T*. *castaneum* and *T*. *confusum*. In both *Tribolium* species, early arrival to competitive arenas resulted in greater population growth relative to late-arriving competitors, thus demonstrating a priority effect. These results contribute to the existing discussion regarding the relative importance of deterministic and stochastic processes in driving community assembly. Deterministic models predict communities will eventually comprise of species which are ecologically matched to the given environment, regardless of the order in which they arrive [38]. In contrast, neutral stochastic models allow for variation in colonization history to result in different communities across similar environments [39,40]. Early arrival allowed both *Tribolium* species to dominate under similar competitive environments, which is consistent with the neutral stochastic model. However, while species identity poorly predicted competitive outcome in priority treatments, *T*. *castaneum* populations appeared to grow larger after following early-arrival than *T*. *confusum*. Additionally, *T*. *confusum* dominated arenas when introduced simultaneously, which could suggest the experimental environment favored competitive performance in this species relative to *T*. *confusum*. Thus, both stochastic and niche-based deterministic processes are relevant to understanding competitive dynamics in *Tribolium*. Adult beetles also exhibited severely reduced survivorship in several treatment groups, which was associated with declining substrate levels. Discussed below are potential mechanisms for the asymmetrical priority effect, and the population collapse.

### Single-species populations

*T*. *confusum* population size was substantially larger in single-species treatments than *T*. *castaneum*. Populations of both species increased roughly four months after founding individuals were introduced to the arenas. This pattern was observed across all treatments, which is unexpected given *Tribolium* developmental time (*i*.*e*., from egg to pupa) in similar experimental conditions is typically 30 days [32]. Adult *Tribolium* are known to cannibalize eggs [25,41], which may have delayed the initial increase of adult numbers. In *T*. *castaneum*, populations also appeared to collapse after month six, which was correlated with reduced quantities of the experimental medium. Adult populations therefore may have been impacted by the combined effects of food limitation and cannibalism between life stages. However, unlike *T*. *castaneum*, single-species populations of *T*. *confusum* did not collapse. It is difficult to predict whether *T*. *confusum* in single-species trials would have persisted beyond the seven month experimental period.

### Immediate and priority competition

When beetles were introduced simultaneously to arenas, *T*. *castaneum* attained higher population sizes than *T*. *confusum*. Successful competition of *T*. *castaneum* during immediate competition which could be explained by this species’ enhanced reproduction in relatively warm and moist environments [35]. All phases of the present experiment proceeded at 26 ± 1°C and 55 ± 5% R.H., which is similar to conditions that favor *T*. *castaneum* over *T*. *confusum* (*e*.*g*., 29°C and 60–70% R.H) [5]. However, peak populations of *T*. *confusum* in single-species populations were higher than *T*. *castaneum*, which is unexpected if *T*. *castaneum* development was unevenly favored by the experimental climate.

Competitive outcome for both species was strongly predicted by priority arrival. When *T*. *confusum* received priority establishment, *T*. *confusum* attained larger populations than *T*. *castaneum*, but performed worse relative to single-species trials. Thus, interspecies interactions appeared to reduce population growth in *T*. *confusum*, despite successful competition overall. Similarly, *T*. *castaneum* was competitively dominant when given priority access to arenas, and average population size was nearly the same as immediate competition and single-species trials. Thus, priority arrival benefited *T*. *castaneum* performance more than *T*. *confusum*. Given the present experimental conditions, *T*. *castaneum* receives a larger interspecific competitive advantage from priority than *T*. *castaneum*. Other studies investigating competition between insect herbivores, have similarly described how priority unevenly improves species’ performance [42,43]. In [42] a native fly species benefited more from priority access to floral resources than an invasive weevil. However, this apparent asymmetry may be explained by how relatively poor the native flies performed in immediate competition and when weevils received priority access. In the present study, humidity levels may have enhanced initial population growth in *T*. *castaneum*, which allowed this species to benefit more from priority than *T*. *confusum*. However, because this study was conducted under the same temperature and humidity levels throughout, it is uncertain whether *T*. *confusum* would have equally benefited from priority had experimental conditions been more favorable. Future study could determine whether the importance of priority arrival in competition is constrained by how much the environment already favors the early arriving species.

Precisely what advantages *T*. *confusum* and *T*. *castaneum* obtain from the two-week period are unclear. Previous research investigating *Tribolium* behavior suggests egg and larval predation is the primary source of interspecies interactions [44–46]. Egg consumption also appears to improve fecundity in *T*. *castaneum* females more than *T*. *confusum* [47,48], which may explain why *T*. *castaneum* populations in immediate and priority competition did not differ from their single-species controls. Thus, colonization priority may allow *Tribolium* to produce enough eggs so as to minimize predation costs from the larval and adult stages of a late-arriving species.

The effect of priority may be enhanced when early arriving species impact their environment in a manner that limits the success of late-arriving species [9]. For instance, developing *Tribolium* populations are known to release toxic metabolites as waste products into the surrounding medium which can disrupt reproductive success of other competitors [49,50]. Chemical conditioning could also explain instances in which the advantage of priority is asymmetrical, as fecundity in *T*. *castaneum* appears to be more negatively impacted by conditioned medium than *T*. *confusum* [51]. In addition, early arriving species likely gain access to unspoiled resources for much longer than competitors. Since the experimental medium was not refreshed during trials, *Tribolium* populations may have become nutrient limited. If true, *Tribolium* benefits from early-arrival by preempting high quality resources, which is a primary mechanism underlying priority effects in plant and animal communities [9].

A final, alternative explanation may involve asymmetrical reproductive interference arising between the two competitors, which in some cases can drive species exclusion more often than resource competition [52]. For instance, interspecies mating attempts by male bean weevils of *Callosobruchus chinensis* reduced the egg production of C. *maculatus* females much more than when *C*. *maculatus* attempted copulation with *C*. *chinensis* females [53]. This asymmetry becomes more severe as the adult population of the reproductively dominant species increases, thus leading to a positive dependent frequency effect. In *Tribolium*, female fecundity in *T*. *castaneum* becomes more inhibited as the density of adult *T*. *confusum* males increases [54], and this decline is not explained by egg cannibalism alone. Thus, early arriving species may reach higher densities sooner, which would limit successful reproduction of competing species. Further research should determine which of these factors is most important for explaining the observed priority effect.

### Population growth and substrate consumption

Adult beetle populations appeared to be limited by available bran substrate, with *T*. *castaneum* populations most strongly impacted. In single-species populations, total bran mass in *T*. *castaneum* trials declined more rapidly than *T*. *confusum* trials, despite *T*. *castaneum* attaining smaller monthly populations.

In all trials, adult survivorship between month six and seven differed across treatments. *T*. *confusum* populations appeared to collapse only in immediate competition trials, and when *T*. *castaneum* adults were introduced two weeks prior. *T*. *confusum* survivorship was near 100% during single-species trials and when given priority arrival, which suggests competitive interactions, in addition to limited food, prevented long-term establishment. In contrast, *T*. *castaneum* populations unexpectedly collapsed in all treatments except when *T*. *confusum* received priority.

Adult survivorship in both species was strongly predicted by total substrate loss, however *T*. *castaneum* was more negatively affected by limited resources than *T*. *confusum*. For both species, adult survivorship was highest when less substrate was consumed. Substrate consumption also increased with population size, which explains why survivorship was highest for both species in arenas with smaller populations, such as when *T*. *confusum* received priority. In competition studies where food is not renewed, coexistence appears to be stable for much longer than in environments where resources are continuously resupplied [55,56]. In *Tribolium*, competitive pressures may reduce population growth in both species, thereby reducing the rate of resource decline [57]. While the present experiment did not control for the effect of food limitation on beetle competition, reduced population size from interspecific interactions may have improved both species’ survivorship by regulating food consumption.

Population collapse in *Tribolium* has been documented by previous competition studies and is typically attributed to excessive cannibalism [5,26]. Cannibalism normally regulates *Tribolium* population growth in high densities [58,59]. However, if substrate volume rapidly declines as it did in the present experiment, encounters between life stages will become more likely, which is expected to increase cannibalistic behavior [60]. Additionally, cannibalism in *T*. *castaneum* may be exacerbated by nutritionally deficient medium, which can lead to the “near-suicide” of populations [27]. In such cases, *T*. *castaneum* could be compensating for nutrients missing from the substrate environment by increasing predation on egg and immature stages. In the present study, the experimental medium contained brewers’ yeast, which can provide the nutrition homogenous grain mixtures may lack [27,61]. However, given that the medium was not refreshed during the experimental period, diminished substrate quality likely contributed to *Tribolium* collapse. Additionally, given that reduced survival was observed in *Tribolium* adults, increased egg or larval cannibalism cannot fully explain the population collapse. These patterns instead suggest that *Tribolium* populations are sensitive to limited resources, and may have exceeded the carrying capacity within the experimental environment.
